# Supplementary material for: The cerebellum is involved in processing of predictions and prediction errors in a fear conditioning paradigm
Source: eLife. 2019 Aug 29;8:e46831. doi: 10.7554/eLife.46831 (PMC6715348; doi:10.7554/eLife.46831)
Supplement: Supplementary file 1. — Table 1, first interval skin conductance responses; Table 2, changes in cerebellar activation across blocks during acquisition and extinction; Table 3, results of cerebellar and whole brain conjunction analyses; Table 4, differences across acquisition contrasts in cerebellar and whole brain activation; Table 5, whole brain activations during acquisition and extinction. [file elife-46831-supp1.docx]

Supplementary Materials

Table of Contents

[Supplementary Table 1: First interval skin conductance responses 2](#_Toc13644103)

[Supplementary Table 2: Changes in cerebellar activation across blocks during acquisition and extinction 3](#_Toc13644104)

[Supplementary Table 3: Cerebellar and whole brain conjunction analyses 4](#_Toc13644105)

[Supplementary Table 4: Differences in cerebellar and whole brain activation 6](#_Toc13644106)

[Supplementary Table 5: Whole brain activations during acquisition and extinction 8](#_Toc13644107)

[References 12](#_Toc13644108)

# Supplementary Table 1: First interval skin conductance responses

Summary of statistical findings (repeated measures ANOVA; post-hoc *t*-tests). CS type = CS+ vs. CS-; phase = acquisition vs. extinction; block = early vs. late.

| **ANOVA** | **contrast** | | | **degrees of freedom** | **F-value** | ***P*** |
| --- | --- | --- | --- | --- | --- | --- |
| acquisition | CS type | | | 1, 21 | 7.34 | 0.013 |
|  | block | | | 1, 21 | 17.80 | < 0.001 |
|  | CS type × block | | | 1, 21 | 0.01 | 0.918 |
| Extinction | CS type | | | 1, 21 | 0.00 | 0.991 |
|  | block | | | 1, 21 | 12.19 | 0.002 |
|  | CS type × block | | | 1, 21 | 0.10 | 0.751 |
| ***t*-tests** | **contrast** |  |  | **degrees of freedom** | **t-value** | ***p*** |
|  | Habituation, CS+ - CS- |  |  | 21 | 1.12 | 0.275 |
|  | Early acquisition, CS+ - CS- |  |  | 21 | 2.60 | 0.017 |
|  | Late acquisition, CS+ - CS- |  |  | 21 | 2.22 | 0.038 |
|  | Early extinction, CS+ - CS- |  |  | 21 | 0.15 | 0.882 |
|  | Late extinction, CS+ - CS- |  |  | 21 | -0.17 | 0.868 |

# Supplementary Table 2: Changes in cerebellar activation across blocks during acquisition and extinction

Main effect of block during acquisition and extinction. Displayed are all clusters of 20 mm^3^ or larger. In each cluster, up to three maxima are listed separated by 8 mm or more.

| **Index** | **Location  (lobule)** | **Side** | **SUIT coordinates / mm** | | | **Cluster size / mm^3^** | | ***p_FWE_*** | **TFCE** |
| --- | --- | --- | --- | --- | --- | --- | --- | --- | --- |
| ***a) CS+ > CS- : Effect of block F-test, TFCE, p < 0.05 FWE corr.*** | | | | | | | | | |
| 1 | VI | Right | 35 | -58 | -28 | 294 | 0.004 | | 28512.3 |
| 2 | Extended cluster | left Crus I (239), left VI (1) | | | | | | | |
|  | Crus I | Left | -43 | -56 | -32 | 240 | 0.006 | | 24891.7 |
|  | Crus I | Left | -35 | -52 | -33 |  | 0.044 | | 16501.8 |
| ***b) no-US post CS+ > no-US post CS- : Effect of block F-test, TFCE, p < 0.05 FWE corr.*** | | | | | | | | | |
| 1 | Extended cluster | left Crus I (776), left VI (311), left Crus II (118) | | | | | | | |
|  | Crus I | Left | -12 | -78 | -32 | 1205 | 0.002 | | 155108 |
|  | VI | Left | -27 | -73 | -25 |  | 0.002 | | 137279 |
|  | VI | Left | -18 | -75 | -25 |  | 0.008 | | 120878 |
| 2 | Extended cluster | left Crus I (400), left VI (250) | | | | | | | |
|  | VI | Left | -33 | -64 | -27 | 650 | 0.002 | | 146662 |
|  | Crus I | Left | -35 | -57 | -31 |  | 0.002 | | 137880 |
|  | Crus I | Left | -40 | -64 | -31 |  | 0.007 | | 123883 |
|  |  |  |  |  |  |  |  | |  |

# Supplementary Table 3: Cerebellar and whole brain conjunction analyses

Cerebellar regions identified using SUIT labels (Cerebellum-SUIT.nii, Diedrichsen, 2006); whole brain regions identified using AAL atlas labels (AAL.nii, Tzourio-Mazoyer et al., 2002). Clusters with 20 or more voxels are included (global null hypothesis, *p* < 0.05 FWE corrected). Up to three maxima per cluster are listed.

| **Index** | **Location** | **Side** | **SUIT coordinates / mm** | | | **Cluster size / mm^3^** | | ***p_FWE_*** | ***t*** |
| --- | --- | --- | --- | --- | --- | --- | --- | --- | --- |
| ***Cerebellar conjunction analysis: US post CS+ > no-US post CS- & CS+ > CS- & no-US post CS+ > no-US post CS- global null hypothesis, FWE corrected p < 0.05*** | | | | | | | | | |
| 1 | Extended cluster | left Crus I (2891), left VI (1715), left Crus II (108), gray matter (27) | | | | | | | |
|  | Crus I | left | -32 | -58 | -34 | 4730 | < 0.001 | | 4.53 |
|  | Crus I | left | -44 | -64 | -29 |  | < 0.001 | | 4.45 |
|  | Crus I | left | -17 | -77 | -27 |  | < 0.001 | | 3.88 |
| 2 | Extended cluster | right Crus I (697), right VI (409) | | | | | | | |
|  | Crus I | right | 39 | -67 | -28 | 1106 | < 0.001 | | 3.26 |
|  | Crus I | right | 48 | -61 | -28 |  | 0.001 | | 2.9 |
|  | VI | right | 30 | -69 | -24 |  | 0.002 | | 2.89 |
| 3 | VIIIb | right | 25 | -56 | -46 | 40 | 0.001 | | 2.98 |
| 4 | Crus II | left | -33 | -62 | -46 | 121 | 0.001 | | 2.94 |
| 5 | gray matter | left | -31 | -53 | -45 | 29 | 0.003 | | 2.82 |
| 6 | VIIb | right | 32 | -58 | -46 | 27 | 0.003 | | 2.79 |
| ***Whole brain conjunction analysis: US post CS+ > no-US post CS- & CS+ > CS- & no-US post CS+ > no-US post CS-  global null hypothesis, FWE corrected p < 0.05*** | | | | | | | | | |
| 1 | Extended cluster | left Insula (1916), left Frontal_Inf_Tri (790), left Frontal_Inf_Oper (738), left Frontal_Inf_Orb (221), outside GM (97), left Rolandic_Oper (64), left Temporal_Pole_Sup (15) | | | | | | | |
|  | Insula | left | -31 | 17 | 4 | 3841 | < 0.001 | | 5.7 |
|  | Frontal_Inf_Tri | left | -42 | 15 | 7 |  | < 0.001 | | 4.6 |
|  | Insula | left | -31 | 26 | -2 |  | < 0.001 | | 4.5 |
| 2 | Extended cluster | right Insula (1770), right Frontal_Inf_Tri (973), right Frontal_Inf_Oper (905), outside GM (802), right Rolandic_Oper (140), right Frontal_Inf_Orb (112), right Putamen (73) | | | | | | | |
|  | Frontal_Inf_Tri | right | 37 | 27 | 2 | 4775 | < 0.001 | | 4.8 |
|  | Frontal_Inf_Oper | right | 43 | 18 | 5 |  | < 0.001 | | 4.7 |
|  | Rolandic_Oper | right | 53 | 6 | 8 |  | < 0.001 | | 3.9 |
| 3 | Extended cluster | left Lob. Crus I (1839), left Lob. VI (1105), left Fusiform (1) | | | | | | | |
|  | Lob. Crus I | left | -32 | -59 | -34 | 2945 | < 0.001 | | 4.7 |
|  | Lob. Crus I | left | -44 | -66 | -28 |  | < 0.001 | | 4.5 |
|  | Lob. Crus I | left | -18 | -77 | -27 |  | < 0.001 | | 3.7 |
| 4 | Extended cluster | right SupraMarginal (2280), right Temporal_Sup (337), right Parietal_Inf (74), right Angular (48) | | | | | | | |
|  | SupraMarginal | right | 53 | -43 | 28 | 2703 | < 0.001 | | 4.3 |
|  | SupraMarginal | right | 60 | -39 | 29 |  | < 0.001 | | 4.2 |
|  | SupraMarginal | right | 54 | -41 | 37 |  | < 0.001 | | 4.1 |
| 5 | Extended cluster | outside GM (310), right Thalamus (12) | | | | | | | |
|  | outside GM | right | 5 | -30 | -8 | 322 | < 0.001 | | 4.2 |
|  | outside GM | right | 7 | -29 | -19 |  | < 0.001 | | 3.7 |
| 6 | Thalamus | right | 11 | -7 | 6 | 260 | < 0.001 | | 4.1 |
| 7 | Extended cluster | right Precentral (391), right Frontal_Mid (301) | | | | | | | |
|  | Precentral | right | 44 | 8 | 50 | 692 | < 0.001 | | 4 |
|  | Precentral | right | 43 | 2 | 39 |  | < 0.001 | | 3.4 |
| 8 | Extended cluster | outside GM (208), left Thalamus (14) | | | | | | | |
|  | outside GM | left | -9 | -24 | -8 | 222 | < 0.001 | | 3.9 |
|  | outside GM | left | -3 | -30 | -13 |  | < 0.001 | | 3.8 |
|  | outside GM | left | -3 | -26 | -3 |  | 0.01 | | 2.9 |
| 9 | Extended cluster | right Supp_Motor_Area (685), left Supp_Motor_Area (396), outside GM (6) | | | | | | | |
|  | Supp_Motor_Area | right | 6 | 15 | 51 | 1087 | < 0.001 | | 3.8 |
|  | Supp_Motor_Area | left | 1 | 17 | 57 |  | < 0.001 | | 3.8 |
|  | Supp_Motor_Area | right | 4 | 12 | 67 |  | < 0.001 | | 3.5 |
| 10 | Extended cluster | right Lob. VI (339), right Lob. Crus I (73) | | | | | | | |
|  | Lob. VI | right | 37 | -61 | -27 | 412 | < 0.001 | | 3.7 |
|  | Lob. VI | right | 26 | -64 | -32 |  | < 0.001 | | 3.4 |
|  | Lob. VI | right | 35 | -69 | -26 |  | 0.004 | | 3.1 |
| 11 | Thalamus | left | -12 | -9 | 7 | 162 | < 0.001 | | 3.6 |
| 12 | outside GM | left | -7 | -16 | -10 | 38 | < 0.001 | | 3.5 |
| 13 | Extended cluster | left SupraMarginal (576), left Parietal_Inf (148), outside GM (58) | | | | | | | |
|  | SupraMarginal | left | -62 | -46 | 32 | 779 | < 0.001 | | 3.5 |
|  | SupraMarginal | left | -60 | -41 | 24 |  | < 0.001 | | 3.5 |
|  | SupraMarginal | left | -53 | -41 | 32 |  | < 0.001 | | 3.4 |
| 14 | Frontal_Sup | right | 28 | 45 | 21 | 88 | < 0.001 | | 3.4 |
| 15 | Frontal_Sup | right | 23 | 52 | 22 | 147 | < 0.001 | | 3.4 |
| 16 | Precentral | left | -39 | -2 | 54 | 68 | < 0.001 | | 3.4 |
| 17 | Lob. Crus II | left | -15 | -75 | -36 | 31 | 0.001 | | 3.3 |
| 18 | Supp_Motor_Area | left | -10 | 5 | 66 | 64 | 0.001 | | 3.3 |
| 19 | Frontal_Sup_Medial | left | 1 | 30 | 33 | 203 | 0.001 | | 3.3 |
|  |  |  |  |  |  |  |  | |  |

# Supplementary Table 4: Differences across acquisition contrasts in cerebellar and whole brain activation

Differences in cerebellar activations comparing the three main acquisition contrasts based on *F*-tests (using TFCE, *p* < 0.05 FWE corrected). Cerebellar regions identified using SUIT labels; whole brain regions using AAL atlas labels. Clusters with 20 or more voxels are included. Up to three maxima per cluster are displayed.

| **Index** | **Location** | **Side** | **SUIT coordinates / mm** | | | **Cluster size / mm^3^** | | ***p_FWE_*** | ***TFCE F*** |
| --- | --- | --- | --- | --- | --- | --- | --- | --- | --- |
| ***Differences in cerebellar activations: US post CS+ > no-US post CS- vs. CS+ > CS- vs. no-US post CS+ > no-US post CS-  TFCE, FWE corrected p < 0.05*** | | | | | | | | | |
| 1 | Extended cluster | left Crus I (1021), left VI (563) | | | | | | | |
|  | VI | left | -26 | -73 | -25 | 1584 | 0.002 | | 755254 |
|  | Crus I | left | -17 | -78 | -25 |  | 0.002 | | 733766 |
|  | Crus I | left | -37 | -63 | -27 |  | 0.017 | | 508636 |
| 2 | I-IV | right | 3 | -54 | -22 | 136 | 0.014 | | 520535 |
| ***Differences in whole brain activations: US post CS+ > no-US post CS- vs. CS+ > CS- vs. no-US post CS+ > no-US post CS-  TFCE, FWE corrected p < 0.05*** | | | | | | | | | |
| 1 | Extended cluster | left Insula (5691), outside GM (843), left Frontal_Inf_Oper (801), left Temporal_Sup (499), left Frontal_Inf_Tri (427), left Frontal_Inf_Orb (304), left Temporal_Pole_Sup (129), left Rolandic_Oper (109), left Putamen (70), left Precentral (52), left Heschl (24) | | | | | | | |
|  | Insula | left | -39 | -4 | -4 | 8949 | < 0.001 | | 3886919 |
|  | Temporal_Sup | left | -39 | -14 | -7 |  | < 0.001 | | 2220965 |
|  | Insula | left | -31 | 17 | 3 |  | < 0.001 | | 1613525 |
| 2 | Extended cluster | right Insula (5594), outside GM (2030), right Frontal_Inf_Oper (1670), right Frontal_Inf_Tri (632), right Rolandic_Oper (557), right Frontal_Inf_Orb (517), right Putamen (315), right Temporal_Pole_Sup (74), right Temporal_Sup (28) | | | | | | | |
|  | outside GM |  | 39 | -3 | -8 | 11417 | < 0.001 | | 2200812 |
|  | Insula | right | 42 | 9 | -6 |  | < 0.001 | | 2126720 |
|  | Insula | right | 40 | -10 | -4 |  | < 0.001 | | 2095510 |
| 3 | Extended cluster | right SupraMarginal (811), right Temporal_Sup (56), outside GM (11), right Rolandic_Oper (6) | | | | | | | |
|  | SupraMarginal | right | 63 | -38 | 27 | 884 | 0.001 | | 1294068 |
|  | SupraMarginal | right | 56 | -33 | 27 |  | 0.008 | | 1024451 |
|  | SupraMarginal | right | 62 | -26 | 22 |  | 0.016 | | 9025467 |
| 4 | outside GM | left | -5 | -29 | -8 | 179 | 0.010 | | 966886 |
| 5 | Extended cluster | left Lob. Crus I (240), left Lob. VI (195) | | | | | | | |
|  | Lob. VI | left | -27 | -74 | -23 | 435 | 0.015 | | 904621 |
|  | Lob. Crus I | left | -19 | -78 | -24 |  | 0.017 | | 881553 |
| 6 | SupraMarginal | left | -55 | -39 | 25 | 244 | 0.018 | | 862433 |
| 7 | outside GM | left | -61 | -37 | 41 | 40 | 0.036 | | 785741 |
| 8 | SupraMarginal | left | -59 | -24 | 17 | 55 | 0.038 | | 776401 |
| 9 | Postcentral | left | -57 | -23 | 25 | 33 | 0.039 | | 772784 |
| 10 | SupraMarginal | right | 64 | -33 | 36 | 37 | 0.042 | | 760703 |
|  |  |  |  |  |  |  |  | |  |

# Supplementary Table 5: Whole brain activations during acquisition and extinction

Displayed are all clusters of 20 mm^3^ or larger. In each cluster up to three maxima are listed separated by 8 mm or more.

| **Index** | **Location** | **Side** | **SUIT coordinates / mm** | | | **Cluster size / mm^3^** | | ***p_FWE_*** | **TFCE** | |
| --- | --- | --- | --- | --- | --- | --- | --- | --- | --- | --- |
| ***US post CS+ > no-US post CS- FWE corrected p < 0.05*** | | | | | | | | | | |
| 1 | Extended cluster | outside GM (80294), left Insula (12830), right Frontal_Mid (12274), right Insula (12057), right SupraMarginal (11987), left Lob. Crus I (10060), left Lob. VI (9432), right Frontal_Inf_Tri (8704), left Cingulum_Mid (8377), right Lob. VI (7744), left SupraMarginal (7569), right Rolandic_Oper (6804), right Cingulum_Mid (6651), right Temporal_Mid (6472), right Temporal_Sup (6419), right Frontal_Inf_Oper (6290), right Supp_Motor_Area (6280), left Parietal_Inf (6124), left Supp_Motor_Area (5999), right Putamen (5904), right Lob. VIII (5843), left Rolandic_Oper (5485), left Calcarine (5359), left Frontal_Inf_Oper (5036), left Temporal_Sup (4941), left Cingulum_Ant (4765), left Lob. VIII (4743), right Frontal_Inf_Orb (4713), left Lob. IV-V (4711), left Lob. Crus II (4613), vermal Lob. IV-V (4533), right Parietal_Inf (4373), left Thalamus (4352), left Precuneus (4172), right Caudate (3976), left Putamen (3966), right Calcarine (3872), right Frontal_Sup_Medial (3740), right Thalamus (3555), right Lob. IV-V (3534), right Cingulum_Ant (3524), left Postcentral (3294), right Frontal_Sup (3091), left Frontal_Inf_Tri (2837), right Lob. IX (2752), left Frontal_Sup_Medial (2619), left Lingual (2361), right Precuneus (2331), left Frontal_Sup (2166), right Lob. Crus I (2113), left Caudate (2106), vermal Lob. VI (1945), left Frontal_Inf_Orb (1856), right Precentral (1738), right Lingual (1552), right Temporal_Pole_Sup (1540), left Lob. IX (1502), vermal Lob. VIII (1445), left Precentral (1378), left Temporal_Pole_Sup (1349), left Lob. VIIb (1280), left Pallidum (1139), vermal Lob. IX (1040), left Temporal_Inf (981), right Postcentral (952), left Heschl (923), right Pallidum (875), right Hippocampus (839), left Paracentralobule (757), right Amygdala (726), left Hippocampus (704), vermal Lob. III (667), right Frontal_Mid_Orb (631), right Angular (554), right Heschl (489), left Amygdala (472), right Temporal_Inf (436), left Paracentralobule (362), left Cuneus (357), right Frontal_Sup_Orb (347), left Fusiform (324), right Fusiform (302), right Parietal_Sup (300), right Lob. Crus II (300), left Lob. III (277), vermal Lob. VII (268), right Lob. VIIb (241), right Olfactory (205), right Lob. III (180), vermal Lob. X (166), vermal Lob. 1_2 (146), left ParaHippocampal (144), left Olfactory (115), right ParaHippocampal (80), left Lob. X (72), right Lob. X (72), left Cingulum_Post (71), right Temporal_Pole_Mid (53), left Frontal_Mid (31), right Cingulum_Post (25), left Temporal_Mid (21), right Rectus (19), right Cuneus (16), left Parietal_Sup (14), left Frontal_Sup_Orb (12), left Occipital_Sup (1) | | | | | | | | |
|  | Insula | right | 42 | -3 | -7 | 390038 | < 0.001 | | 18195.4 | |
|  | Insula | left | -37 | -10 | -5 |  | < 0.001 | | 18164.9 | |
|  | Insula | right | 41 | -11 | -9 |  | < 0.001 | | 17925.9 | |
| 2 | Extended cluster | left Precuneus (833), left Parietal_Sup (92), left Cuneus (60), left Occipital_Sup (34) | | | | | | | | |
|  | Precuneus | left | -9 | -74 | 42 | 1019 | 0.029 | | 3199.4 | |
|  | Cuneus | left | -3 | -80 | 40 |  | 0.047 | | 2779.1 | |
| 3 | Extended cluster | left Parietal_Sup (1146), left Postcentral (577), left Precuneus (50), outside GM (26) | | | | | | | | |
|  | Postcentral | left | -25 | -43 | 67 | 1799 | 0.032 | | 3118.3 | |
|  | Parietal_Sup | left | -18 | -49 | 69 |  | 0.033 | | 3102.4 | |
|  | Postcentral | left | -25 | -37 | 73 |  | 0.044 | | 2855.1 | |
| 4 | Extended cluster | left Frontal_Mid (2317), left Frontal_Inf_Tri (1775), left Frontal_Sup (235), outside GM (91) | | | | | | | | |
|  | Frontal_Inf_Tri | left | -46 | 42 | 4 | 4418 | 0.033 | | 3102.1 | |
|  | Frontal_Mid | left | -31 | 49 | 24 |  | 0.036 | | 3003.0 | |
|  | Frontal_Mid | left | -45 | 39 | 21 |  | 0.036 | | 3002.0 | |
| 5 | outside GM |  | 33 | -36 | 18 | 52 | 0.037 | | 2997.7 | |
| 6 | outside GM |  | 12 | -92 | -14 | 34 | 0.037 | | 2984.9 | |
| 7 | Extended cluster | left Temporal_Mid (741), outside GM (17) | | | | | | | | |
|  | Temporal_Mid | left | -56 | -58 | 3 | 758 | 0.037 | | 2981.6 | |
|  | outside GM |  | -46 | -53 | 2 |  | 0.040 | | 2939.3 | |
|  | Temporal_Mid | left | -48 | -54 | 10 |  | 0.041 | | 2908.4 | |
| 8 | Frontal_Mid | left | -35 | 43 | 1 | 53 | 0.049 | | 2758.8 | |
| ***CS+ > CS- FWE corrected p < 0.05*** | | | | | | | | | | |
| 1 | Extended cluster | left Supp_Motor_Area (3488), left Cingulum_Mid (2817), right Supp_Motor_Area (2769), right Cingulum_Mid (1757), left Cingulum_Ant (701), left Frontal_Sup_Medial (684), outside GM (554), right Frontal_Sup_Medial (228), right Cingulum_Ant (209), left Frontal_Sup (58), right Frontal_Sup (53), left Precentral (3) | | | | | | | | |
|  | Supp_Motor_Area | right | 2 | 10 | 61 | 13321 | 0.013 | | 3708.7 | |
|  | Cingulum_Ant | left | 1 | 27 | 29 |  | 0.013 | | 3676.5 | |
|  | Supp_Motor_Area | left | -3 | 11 | 54 |  | 0.014 | | 3631.8 | |
| 2 | Extended cluster | right SupraMarginal (1153), right Temporal_Sup (5), right Angular (4) | | | | | | | | |
|  | SupraMarginal | right | 61 | -41 | 27 | 1162 | 0.016 | | 3519.4 | |
|  | SupraMarginal | right | 53 | -40 | 29 |  | 0.020 | | 3343.3 | |
|  | SupraMarginal | right | 50 | -40 | 37 |  | 0.039 | | 2764.9 | |
| 3 | Extended cluster | left Insula (1674), left Frontal_Inf_Tri (565), left Frontal_Inf_Orb (155), left Frontal_Inf_Oper (144), outside GM (18) | | | | | | | | |
|  | Insula | left | -39 | 15 | 7 | 2556 | 0.018 | | 3442.6 | |
|  | Insula | left | -30 | 17 | 7 |  | 0.018 | | 3418.6 | |
|  | Insula | left | -29 | 27 | -2 |  | 0.022 | | 3240.4 | |
| 4 | Extended cluster | right Lob. VI (729), right Lob. Crus I (332) | | | | | | | | |
|  | Lob. VI | right | 35 | -48 | -30 | 1061 | 0.022 | | 3252.6 | |
|  | Lob. VI | right | 32 | -60 | -24 |  | 0.025 | | 3113.8 | |
|  | Lob. Crus I | right | 39 | -58 | -32 |  | 0.028 | | 3016.2 | |
| 5 | Extended cluster | right Insula (1464), right Frontal_Inf_Tri (899), outside GM (745), right Frontal_Inf_Oper (369), right Frontal_Inf_Orb (164), right Putamen (1) | | | | | | | | |
|  | Frontal_Inf_Tri | right | 39 | 29 | 1 | 3642 | 0.023 | | 3214.5 | |
|  | Insula | right | 34 | 22 | 13 |  | 0.023 | | 3190.4 | |
|  | Insula | right | 30 | 27 | 3 |  | 0.024 | | 3148.5 | |
| 6 | outside GM |  | -6 | -29 | -7 | 200 | 0.023 | | 3210.8 | |
| 7 | outside GM |  | 9 | -4 | 6 | 280 | 0.024 | | 3138.4 | |
| 8 | Extended cluster | outside GM (182) |  |  |  |  |  | |  | |
|  | outside GM |  | 6 | -28 | -7 | 182 | 0.026 | | 3062.1 | |
|  | outside GM |  | 9 | -27 | -18 |  | 0.049 | | 2596.5 | |
| 9 | Extended cluster | left Lob. Crus I (480), left Lob. VI (116) | | | | | | | | |
|  | Lob. Crus I | left | -44 | -59 | -32 | 596 | 0.032 | | 2915.4 | |
|  | Lob. Crus I | left | -37 | -54 | -34 |  | 0.034 | | 2887.5 | |
|  | Lob. Crus I | left | -31 | -60 | -33 |  | 0.045 | | 2655.9 | |
| 10 | Extended cluster | left SupraMarginal (226) | | | | | | | | |
|  | SupraMarginal | left | -54 | -41 | 31 | 226 | 0.046 | | 2636.7 | |
|  | SupraMarginal | left | -61 | -46 | 32 |  | 0.049 | | 2573.9 | |
| ***Acquisition – no-US post CS+ > no-US post CS- FWE corrected p < 0.05*** | | | | | | | | | |  |
| 1 | Extended cluster | right Frontal_Mid (11734), outside GM (8351), right Frontal_Inf_Oper (5575), right Frontal_Inf_Tri (5442), right Insula (4346), right Frontal_Inf_Orb (2531), right Putamen (2180), right Frontal_Sup (2001), right Precentral (952), right Thalamus (481), right Caudate (458), right Rolandic_Oper (390), right Frontal_Mid_Orb (327), right Pallidum (278), right Frontal_Sup_Orb (224), right Temporal_Pole_Sup (119), left Thalamus (57), right Olfactory (3) | | | | | | | | |
|  | Insula | right | 36 | 20 | 0 | 45449 | 0.001 | | 4926.3 | |
|  | Frontal_Inf_Orb | right | 33 | 24 | -11 |  | 0.001 | | 4895.6 | |
|  | Frontal_Inf_Tri | right | 50 | 20 | 4 |  | 0.001 | | 4781.1 | |
| 2 | Extended cluster | left Lob. Crus I (6445), left Lob. VI (3064), left Lob. Crus II (773), outside GM (119), left Fusiform (14), left Lob. VIII (4) | | | | | | | | |
|  | Lob. Crus I | left | -24 | -77 | -26 | 10419 | 0.001 | | 4685.0 | |
|  | Lob. Crus I | left | -35 | -75 | -24 |  | 0.001 | | 4649.6 | |
|  | Lob. Crus I | left | -15 | -79 | -22 |  | 0.001 | | 4634.5 | |
| 3 | Extended cluster | left Insula (2780), left Frontal_Inf_Oper (1231), left Frontal_Inf_Tri (841), outside GM (642), left Putamen (499), left Frontal_Inf_Orb (424), left Rolandic_Oper (104), left Temporal_Pole_Sup (47), left Precentral (31) | | | | | | | | |
|  | Insula | left | -31 | 18 | 3 | 6599 | 0.002 | | 4254.5 | |
|  | Insula | left | -30 | 22 | -7 |  | 0.003 | | 4117.3 | |
|  | Putamen | left | -27 | 11 | 4 |  | 0.006 | | 3672.0 | |
| 4 | Extended cluster | left Frontal_Sup_Medial (3900), right Supp_Motor_Area (2877), right Frontal_Sup_Medial (2070), left Supp_Motor_Area (1762), right Cingulum_Mid (592), outside GM (290), right Frontal_Sup (216), left Cingulum_Ant (115), right Cingulum_Ant (104), left Cingulum_Mid (95), left Frontal_Sup (57) | | | | | | | | |
|  | Frontal_Sup_Medial | left | -5 | 27 | 49 | 12078 | 0.006 | | 3607.7 | |
|  | Supp_Motor_Area | right | 6 | 19 | 58 |  | 0.011 | | 3225.5 | |
|  | Supp_Motor_Area | left | 0 | 24 | 55 |  | 0.012 | | 3212.4 | |
| 5 | Extended cluster | left Parietal_Inf (465), outside GM (259), left SupraMarginal (83) | | | | | | | | |
|  | outside GM |  | -60 | -46 | 41 | 807 | 0.018 | | 2923.1 | |
|  | outside GM |  | -63 | -51 | 33 |  | 0.023 | | 2819.0 | |
|  | outside GM |  | -60 | -40 | 47 |  | 0.038 | | 2539.2 | |
| 6 | Lob. IV-V | vermal | 1 | -55 | -22 | 139 | 0.030 | | 2661.4 | |
| 7 | Extended cluster | right SupraMarginal (178), right Angular (173) | | | | | | | | |
|  | Angular | right | 52 | -50 | 26 | 351 | 0.038 | | 2534.1 | |
|  | SupraMarginal | right | 60 | -49 | 26 |  | 0.041 | | 2482.2 | |
| 8 | Extended cluster | right SupraMarginal (484), right Parietal_Inf (290), outside GM (7) | | | | | | | | |
|  | SupraMarginal | right | 60 | -47 | 42 | 781 | 0.040 | | 2507.7 | |
|  | SupraMarginal | right | 54 | -43 | 38 |  | 0.041 | | 2472.1 | |
|  | SupraMarginal | right | 62 | -34 | 39 |  | 0.042 | | 2461.7 | |
| 9 | outside GM |  | -4 | -8 | -7 | 4 | 0.041 | | 2476.7 | |
| 10 | Cingulum_Ant | right | 12 | 17 | 27 | 38 | 0.046 | | 2431.0 | |
| 11 | SupraMarginal | right | 65 | -40 | 27 | 24 | 0.049 | | 2384.4 | |
|  |  |  |  |  |  |  |  | |  | |

# References

Diedrichsen, J., 2006. A spatially unbiased atlas template of the human cerebellum. Neuroimage. 33**,** 127-38.

Tzourio-Mazoyer, N., et al., 2002. Automated anatomical labeling of activations in SPM using a macroscopic anatomical parcellation of the MNI MRI single-subject brain. Neuroimage. 15**,** 273-89.
